# Two-color fluorescence-guided surgery for head and neck cancer resections

**DOI:** 10.1117/1.JBO.30.S1.S13707

**Published:** 2024-10-29

**Authors:** Dani A. Szafran, Nourhan A. Shams, Antonio Montaño, Syed Zaki Husain Rizvi, Adam W.G. Alani, Kimberley S. Samkoe, Lei G. Wang, Summer L. Gibbs

**Affiliations:** a Oregon Health & Science University, Biomedical Engineering Department, Portland, Oregon, United States; b Oregon State University, Department of Pharmaceutical Science, College of Pharmacy, Portland, Oregon, United States; c Dartmouth College, Thayer School of Engineering, Hanover, New Hampshire, United States; d Dartmouth College, Geisel School of Medicine, Hanover, New Hampshire, United States; e Oregon Health & Science University, Knight Cancer Institute, Portland, Oregon, United States

**Keywords:** fluorescence-guided surgery, near-infrared, nerve imaging, nerve-specific fluorophore, cancer-specific Affibody

## Abstract

**Significance:**

Head and neck squamous cell carcinoma (HNSCC) has the sixth highest incidence worldwide, with >650,000 cases annually. Surgery is the primary treatment option for HNSCC, during which surgeons balance two main goals: (1) complete cancer resection and (2) preservation of normal tissues to ensure post-surgical quality of life. Unfortunately, these goals are not synergistic, where complete cancer resection is often limited by efforts to preserve normal tissues, particularly nerves, and reduce life-altering comorbidities.

**Aim:**

Currently, no clinically validated technology exists to enhance intraoperative cancer and nerve recognition. Fluorescence-guided surgery (FGS) has successfully integrated into clinical medicine, providing surgeons with real-time visualization of important tissues and complex anatomy, where FGS imaging systems operate almost exclusively in the near-infrared (NIR, 650 to 900 nm). Notably, this spectral range permits the detection of two NIR imaging channels for spectrally distinct detection.

**Approach:**

Herein, we evaluated the utility of spectrally distinct NIR nerve- and tumor-specific fluorophores for two-color FGS to guide HNSCC surgery. Using a human HNSCC xenograft murine model, we demonstrated that facial nerves and tumors could be readily differentiated using these nerve- and tumor-specific NIR fluorophores.

**Results:**

The selected nerve-specific fluorophore showed no significant difference in nerve specificity and off-target tissue fluorescence in the presence of xenograft head and neck tumors. Co-administration of two NIR fluorophores demonstrated successful tissue-specific labeling of nerves and tumors in spectrally distinct NIR imaging channels.

**Conclusions:**

We demonstrate a comprehensive FGS tool for cancer resection and nerve sparing during HNSCC procedures for future clinical translation.

## 1 Introduction

Head and neck squamous cell carcinoma (HNSCC) is the sixth most prevalent cancer type, with >650,000 cases diagnosed annually worldwide.^1^^,^^2^ Surgical cancer resection remains the preferred treatment for HNSCC,^3^^,^^4^ where the surgeon balances two main challenges: (1) complete surgical resection and (2) preservation of nerves to reduce pain and maintain function. The most important factor for predicting long-term survival following surgery is the completeness of cancer resection.^5^^–^^7^ However, identifying tumors and normal tissues during surgery remains challenging, and positive surgical margins occur in 15% to 30% of patients, resulting in poor outcomes and additional therapy.^8^^,^^9^ Furthermore, additional malignancies are often present and undetected during surgery, representing the second leading cause of death in HNSCC patients.^10^ Secondary to cancer treatment, surgical outcomes are plagued by intraoperative nerve damage, causing lasting pain and loss of function, significantly affecting patient postoperative quality of life. Important cranial, spinal, and facial nerves are small, and translucent structures often buried within tissue, making them difficult to consistently identify and visualize, leading to nerve damage.^11^^–^^14^ As a result, a number of comorbidities, including severe pain, dysphagia, and jaw and sensory dysfunction, are experienced by >40% of patients.^15^ Unfortunately, achieving complete cancer resection while simultaneously preserving important nerves in the head and neck region remains difficult, where efforts to preserve normal anatomy and function limit cancer resection margins. Thus, current intraoperative cancer and nerve detection techniques, generally consisting of preoperative imaging, biopsy, intraoperative neuromonitoring, and visual inspection, fall far short of meeting the clinical need.^16^

To improve intraoperative nerve and tumor identification, tools to enable real-time tissue visualization are under development including the clinical translation of fluorescence-guided surgery (FGS). To employ FGS, clinicians administer a fluorescent contrast agent prior to surgery that accumulates in or marks tissues of interest (e.g., vessels, tumors, and nerve) and deploy specialized intraoperative instruments capable of real-time imaging of the contrast agent distribution in the surgical field. FGS offers real-time imaging using clinical wide-field and laparoscopic FGS vision systems that are capable of imaging near-infrared (NIR) wavelengths (650 to 900 nm). In the NIR spectral range, endogenous tissue chromophore absorbance, autofluorescence, and scatter fall to local minima, providing a low background signal upon which contrast-enhanced tissues can be highlighted at up to centimeter depths. There are currently >125 ongoing clinical trials for FGS contrast agents.^17^ Notably, the first molecularly targeted contrast agents received recent Food and Drug Administration (FDA) approval, including pafolacianine to aid in the resection of ovarian (2021) and lung (2022) cancers as well as pegulicianine (2024) to aid in the resection of breast cancer, further cementing the utility of FGS to aid in precision surgery.^18^^–^^22^ Although the overall goal of FGS is to aid in improved identification of tissues to be removed (e.g., tumors) and those to be preserved (e.g., nerves), no clinical study has yet utilized dual-targeted contrast agents. However, preclinical studies have shown the potential power of two color FGS strategies to visualize nerve and tumor tissues, where a visible nerve-specific probe and red-shifted tumor-targeted probe demonstrated the ability to specifically highlight both structures.^2^

**Fig. 1:**
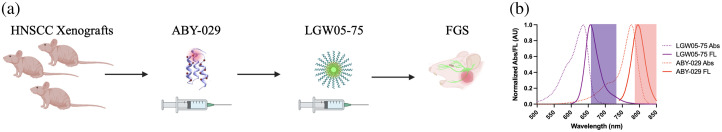
Study overview and contrast agent spectral properties. (a) HNSCC was modeled using cohorts of Detroit 562 tumors implanted next to the facial nerves. The tumors were highlighted using EGFR-targeted ABY-029 followed by the administration of micelle encapsulated LGW05-75 to specifically highlight the facial nerves, enabling two-color FGS. (b) Normalized absorbance and emission spectra of LGW05-75 (purple) and ABY-029 (red) with the emission filters used for fluorescence image collection demonstrate the spectral separation of the nerve- and tumor-specific fluorophores.

To facilitate NIR nerve-specific fluorescence imaging, we developed a library of oxazine-based fluorophores, where a lead fluorophore, LGW05-75, was selected for the two-color FGS studies herein. LGW05-75 was selected for this work due to its bright, NIR fluorescent signal; high nerve specificity; and favorable pharmacokinetic (PK) properties.^23^ Previous work with LGW05-75 showed strong nerve specificity following systemic administration in a laboratory-based, co-solvent formulation composed of dimethyl sulfoxide, Kolliphor ethylene oxide (EL), and serum permitting solubility for intravenous administration.^23^^,^^24^ To enable future clinical translation, a previously developed, FDA-approved micelle-based formulation was evaluated to solubilize LGW05-75 for systemic administration.^25^^,^^26^ Successful formulation of LGW05-75 in the distearyl-phosphatiylethanolamine-PEG2000 (DSPE-PEG) micelles validated this formulation for similarly structured oxazine probes. Herein, the DSPE-PEG-formulated LGW05-75 was combined with the spectrally distinct synthetic Affibody molecule labeled with IRDye 800CW (ABY-029) that targets the epidermal growth factor receptor (EGFR).^27^^–^^29^ This probe is currently in early-phase clinical trials to evaluate safety and utility to highlight a variety of EGFR overexpressing cancers including head and neck pathologies. In this study, the ability to combine ABY-029 and LGW05-75 was investigated, where these two contrast agents were hypothesized to have substantial clinical compatibility due to their tissue specificity, spectral separation, and similar pharmacokinetic profiles (Fig. 1). The overall goal of the current studies was to evaluate the utility of co-administration of NIR nerve- and tumor-targeted contrast agents as a complete FGS solution to enable real-time identification and visualization of diseased and normal tissues enabling precision surgery for cancer patients.

## 2 Materials and Methods

### 2.1 Fluorescent Contrast Agents

A previously developed NIR nerve-specific fluorophore, LGW05-75, was used to provide nerve-specific contrast.^23^ LGW05-75 was formulated in DSPE-PEG micelles to increase solubility as previously described.^25^^,^^26^ LGW05-75 was selected from a previously synthesized library of oxazine-based fluorophores, where it was highlighted as a lead NIR fluorophore [excitation max = 640 nm, emission max = 665 nm, and molecular weight (MW)=369.42  g/mol].^23^ A previously developed tumor-targeting Affibody molecule (MW≈8  kDa, Affibody Medical, Solna, Sweden) targeted to EGFR and conjugated to the NIR fluorophore IRDye 800CW (LI-COR Biosciences, Lincoln, Nebraska, United States) was used to provide tumor-specific contrast.^27^ The NIR-labeled anti-EGFR Affibody, termed ABY-029, was selected due to its safety profile in early-phase clinical trials.^27^^–^^29^

### 2.2 Mice

All animal work was approved by the Institutional Animal Care and Use Committee (IACUC) at Oregon Health and Science University. All mice were housed and treated in accordance with an IACUC-approved protocol. Mixed male and female (50/50) CD-1 mice (n=10) were used to quantify the PK profile of DSPE-PEG micelle–formulated LGW05-75. Mixed male and female (50/50) athymic nude mice (n=20) were used to quantify the LGW05-75 PK profile in the context of tumors, enabling nerve-to-tumor contrast quantification. Mixed male and female (50/50) athymic nude mice (n=20) were used to evaluate multi-color nerve and tumor imaging using LGW05-75 and ABY-029, respectively. At the end of the imaging study, mice were humanely euthanized using carbon dioxide inhalation as the primary method and cervical dislocation as the secondary method of euthanasia.

### 2.3 Cell Culture and Tumor Implantation

The human epithelial pharyngeal carcinoma cell line, Detroit 562, was purchased from American Type Culture Collection (ATCC; Manassas, Virginia, United States). The Detroit 562 cell line was cultured in Eagle’s minimum essential medium (Corning, Glendale, Arizona, United States) formulated with 10% fetal bovine serum (FBS, Seradigm, Sanborn, New York, United States) and 1% penicillin-streptomycin (Thermo Fisher Scientific, Waltham, Massachusetts, United States). Cells were maintained at 37°C and 5% ${\mathrm{CO}}_{2}$. The cells were grown to ∼80% to 90% confluence for xenograft implantation. Detroit 562 cells were implanted at a concentration of $1\times 10^{6}\text{ cells}/100\;\mu L$. Athymic nude mice were anesthetized using 2% isoflurane. A 25-gauge needle was used to implant bilaterally into the cheek area to grow near the buccal and marginal facial nerves. Tumors were monitored and not allowed to grow larger than $0.75\;{\mathrm{cm}}^{3}$ as per the approved IACUC protocol. Tumors were used for imaging studies at sizes ranging from 0.5 to $0.75\;{\mathrm{cm}}^{3}$.

### 2.4 Imaging and Quantification

All images were collected using a custom-built, previously reported small animal imaging system capable of real-time color and fluorescence imaging.^23^^,^^25^^,^^26^^,^^30^^–^^32^ Briefly, the imaging system consisted of a QImaging EXi Blue monochrome camera (Surrey, British Columbia, Canada) for fluorescence detection with a removable Bayer filter permitting the collection of co-registered color and fluorescence images. A PhotoFluor II light source (89 North, Burlington, Vermont, United States) was focused onto the surgical field through a liquid light guide and used unfiltered for white light illumination. For LGW05-75 fluorescence imaging, the PhotoFluor II was filtered with a 620±30-nm bandpass excitation filter. The resulting fluorescence was collected with a 700±37.5-nm bandpass emission filter. For ABY-029 fluorescence imaging, the PhotoFluor II was filtered with a 740±20-nm bandpass excitation filter. The resulting fluorescence was collected with a 780-nm-long pass emission filter. Camera and light source positions were unchanged throughout the imaging studies, which allowed for a quantitative comparison of fluorescence intensities. Camera exposure times ranged from 10 to 1000 ms, where all images were normalized for exposure time. Tissue fluorescence intensities were quantified using custom-written MATLAB code permitting region of interest (ROI) selection for nerves, muscle, and tumor tissues on the white light images but blinded to the fluorescence images. The white light image–selected ROIs were superimposed on the matched fluorescence images enabling unbiased quantification of tissue intensities, which were used to calculate nerve-to-muscle (N/M), nerve-to-tumor (N/T), tumor-to-muscle (T/M), and tumor-to-nerve (T/N) signal-to-background ratios (SBRs).

### 2.5 Pharmacokinetic Profile of DSPE-PEG Micelle–Encapsulated LGW05-75

The PK profile of DSPE-PEG micelle–formulated LGW05-75 was quantified in non-tumor-bearing CD-1 mice and athymic nude mice bearing Detroit 562 tumors. CD-1 and nude mice were fed a chlorophyll-free diet (Animal Specialties, Quakertown, Pennsylvania, United States) for 7 days prior to imaging studies. The CD-1 or tumor-bearing nude mice were injected with DSPE-PEG micelle–formulated LGW05-75 at a dose of 7.5  μmol/kg intravenously in the lateral tail vein. The PK profile of DSPE-PEG-formulated LGW05-75 was evaluated 0.5, 1, 2, 4, and 6 h after intravenous (IV) administration (n=10 CD-1 mice, two mice per time point, 50/50, male/female; n=20 nude mice, four mice per time point, 50/50 male/female). The brachial plexus and facial nerves of the CD-1 mice were exposed and imaged to quantify LGW05-75 biodistribution. The marginal and buccal facial nerves as well as the brachial plexus region of the tumor-bearing nude mice were exposed and imaged for LGW05-75 fluorescence intensity. Six tumor-bearing nude mice (50/50, male/female) were also imaged 4 h after vehicle injection (i.e., blank DSPE-PEG micelles) to quantify nerve and tumor autofluorescence. A one-way analysis of variance (ANOVA) was completed to determine the optimal imaging time point for LGW05-75 nerve imaging after IV administration in CD-1 and tumor-bearing nude mice.

### 2.6 Co-administration of DSPE-PEG Micelle–Encapsulated LGW05-75 and ABY-029

Following completion of the PK study for LGW05-75, the optimal administration to imaging time point in the buccal region was found to be between 2 and 4 h. Previous studies showed the optimal administration to imaging time point for ABY-029 started at 4 h.^28^ Using 4 h as the optimal time point for both LGW05-75 and ABY-029, n=20 nude mice (50/50, male/female) bearing Detroit 562 tumors were co-administered 7.5  μmol/kg of LGW05-75 and 15 nmol of ABY-029 via lateral tail vein injection. Imaging was completed 4 h after systemic administration as a proof of concept that nerves and tumors could be readily visualized using these targeted, spectrally distinct probes at the same time point. An unpaired parametric t-test with Welch’s correction was used to determine if there was a statistically significant difference among LGW05-75 administered alone at 4 h versus when it was co-administered with ABY-029 in tumor-bearing mice.

## 3 Results

### 3.1 DSPE-PEG Micelle–Encapsulated LGW05-75 Nerve PK Profile

**Fig. 2:**
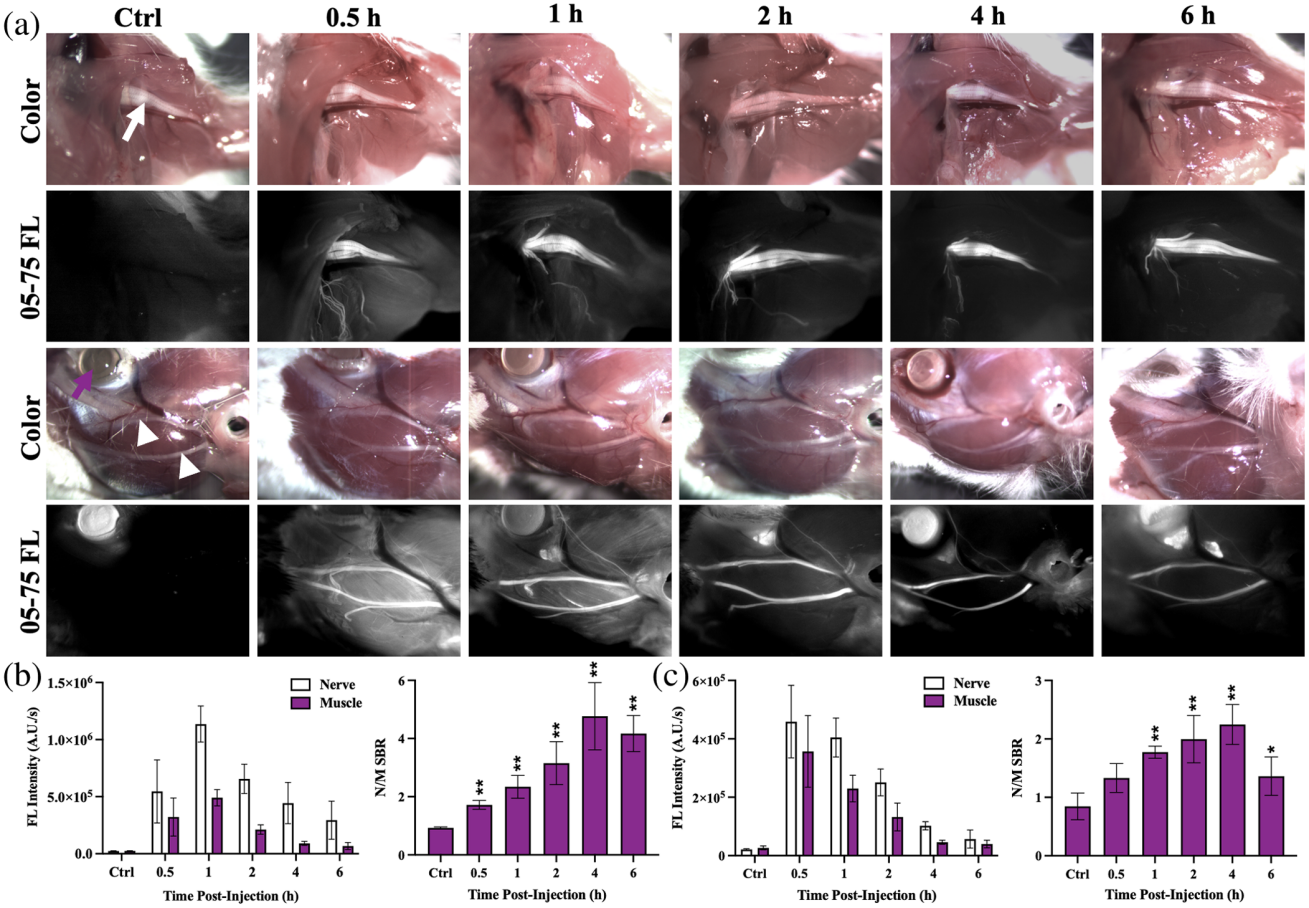
PK profile of DSPE-PEG micelle–formulated LGW05-75 in CD-1 mice. (a) Brachial plexus (top, white arrow, ∼1  mm diameter)^32^ and facial (bottom, white arrowheads, ∼0.1  mm diameter)^33^ nerve color and fluorescence images from representative CD-1 mice at 0.5, 1, 2, 4, and 6 h after intravenous (IV) administration of DSPE-PEG micelle–formulated LGW05-75. A representative control (Ctrl), vehicle-injected mouse with its brachial plexus and facial nerves is also shown. The eye (purple arrow) showed variable fluorescence intensity, which was likely autofluorescence because it was also seen in the control group. Quantification of the nerve and muscle tissue fluorescence (FL) intensities enabled the calculation of the N/M SBR for the (b) brachial plexus and (c) facial nerves. Data were compared with the control autofluorescence to determine the significance of the N/M SBR using a one-way ANOVA not assuming sphericity with the Geisser–Greenhouse correction and an uncorrected Fisher’s least significance difference (LSD) multiple comparison test, where *P<0.05 and **P<0.01.

The NIR nerve-specific fluorophore, LGW05-75, was formulated with the previously optimized DSPE-PEG micellar dispersion^25^^,^^26^ to enable quantification of the PK profile. CD-1 mice were administered the DSPE-PEG micelle–encapsulated LGW05-75 intravenously 0.5, 1, 2, 4, or 6 h prior to fluorescence imaging. Nerve-specific contrast was visible in the brachial plexus as well as facial nerves as early as 0.5 h after systemic administration of DSPE-PEG micelle–formulated LGW05-75. Some fluorescence signals in the surrounding muscle in both the brachial plexus and buccal regions were visible at the early imaging time points [i.e., 0.5 and 1 h; Fig. 2(a)]. By 2 to 4 h post-administration, there was high nerve-to-muscle SBR [Figs. 2(b) and 2(c)]. The nerve and muscle tissue fluorescence intensities steadily decreased over the evaluated 6 h time period post-systemic administration. The brachial plexus nerve showed significantly higher N/M SBR versus control tissue autofluorescence throughout the 6-h imaging period [Fig. 2(b)]. Notably, the facial nerves showed significantly higher N/M SBR 1 to 6 h after systemic administration [Fig. 2(c)]. Both the brachial plexus and facial nerve PK profiles showed peak N/M SBR 4 h after systemic administration.

### 3.2 DSPE-PEG Micelle–Encapsulated LGW05-75 Nerve-to-Tumor PK Profile

**Fig. 3:**
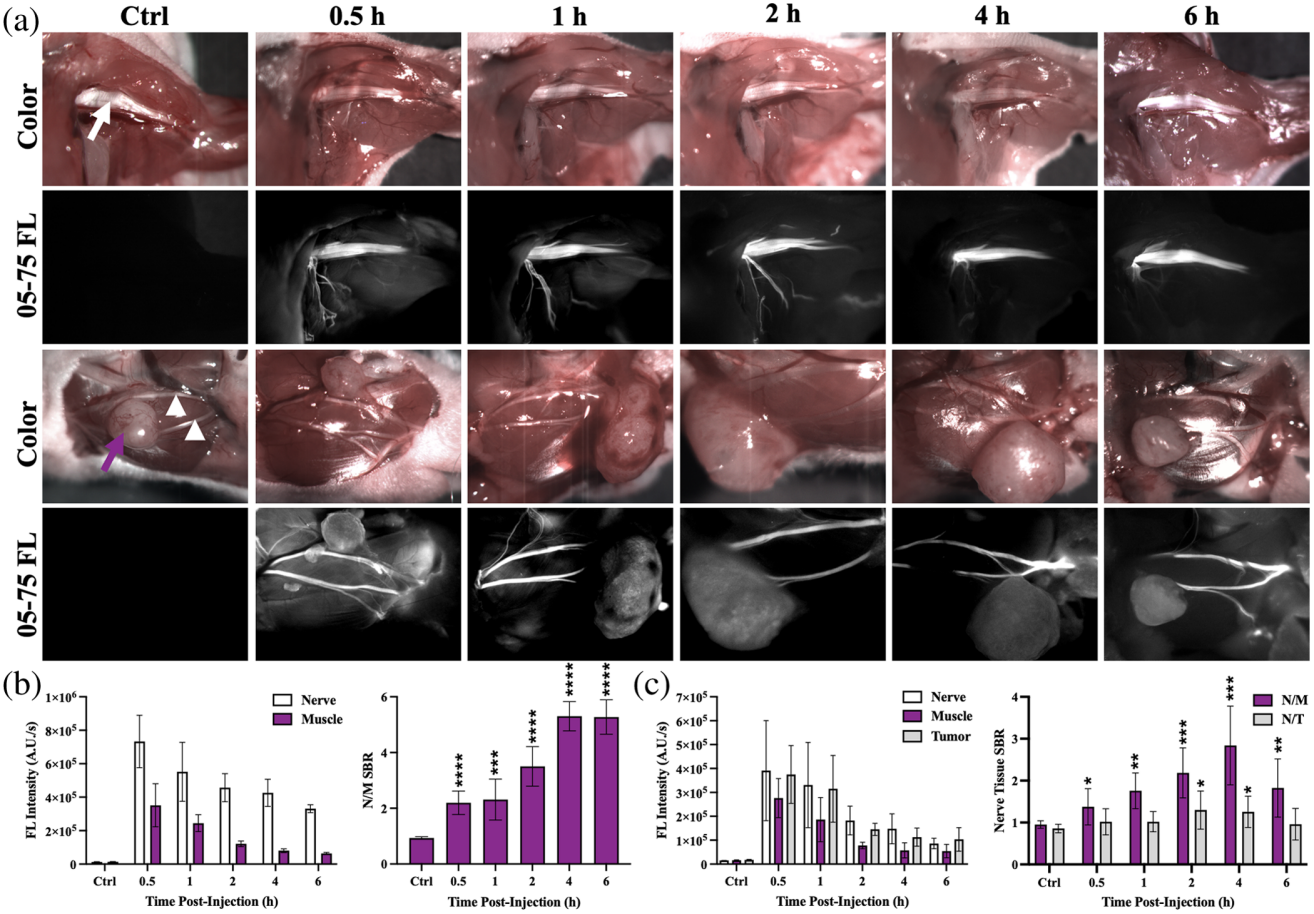
PK profile of DSPE-PEG micelle–encapsulated LGW05-75 in tumor-bearing nude mice. (a) Brachial plexus (top, white arrow) and facial (bottom, white arrowheads) nerve color and fluorescence images from representative athymic nude mice bearing a Detroit 562 tumor (purple arrow) at 0.5, 1, 2, 4, and 6 h after IV administration of DSPE-PEG micelle–formulated LGW05-75. A representative Ctrl, vehicle-injected mouse with its brachial plexus and facial nerves is also shown. Quantification of the nerve and muscle tissue fluorescence intensities enabled the calculation of the N/M signal-to-background ratio (SBR) for the (b) brachial plexus and (c) facial nerves. Statistical analysis was completed to determine the optimal time point for LGW05-75 imaging in the presence of a tumor by comparing data from the 0.5-, 1-, 2-, 4-, and 6-h time points to the control, vehicle-injected group. Data were compared with a one-way ANOVA not assuming sphericity with the Geisser–Greenhouse correction followed by an uncorrected Fisher’s LSD multiple comparisons test, where *P<0.05, **P<0.01, ***P<0.001, and ****P<0.0001.

The PK profile of DSPE-PEG micelle–encapsulated LGW05-75 was evaluated in tumor-bearing mice, where the tumors were grown in the buccal area to enable ready quantification of tumor, nerve, and surrounding normal tissue fluorescence. Tumor-bearing mice were administered the DSPE-PEG micelle–encapsulated LGW05-75 intravenously 0.5, 1, 2, 4, or 6 h prior to fluorescence imaging. Similar to the PK study of LGW05-75 in non-tumor bearing animals (Fig. 2), some muscle tissue uptake was seen at the early time points post-administration [i.e., 0.5 and 1 h; Fig. 3(a)]. The nerve and muscle tissue fluorescence intensity steadily decreased over the evaluated 6 h time period post-systemic administration [Figs. 3(b) and 3(c)]. The brachial plexus nerve showed significantly higher N/M SBR versus control tissue autofluorescence throughout the 6-h imaging period [Fig. 3(b)]. In the buccal region of interest, all time points showed significant N/M SBR compared with control tissue autofluorescence. The buccal, marginal, and zygomatic facial nerves were readily visible at the 4 h time point, which had the highest N/M SBR [Fig. 3(c)]. Although there was clear nerve specificity at the 2-, 4-, and 6-h time points [Fig. 3(a)], the 4-h time point showed the highest SBR in both the brachial plexus and facial nerve regions [Figs. 3(b) and 3(c)]. The nerve-to-tumor contrast was found to be significant at 2 and 4 h post-administration [Fig. 3(c)]. The optimal imaging time point for DSPE-PEG micelle–encapsulated LGW05-75 was determined to be 4 h after systemic administration based on the quantified N/M SBR.

### 3.3 Nerve and Tumor Highlighting Using Co-administration of DSPE-PEG Micelle–Encapsulated LGW05-75 and ABY-029

**Fig. 4:**
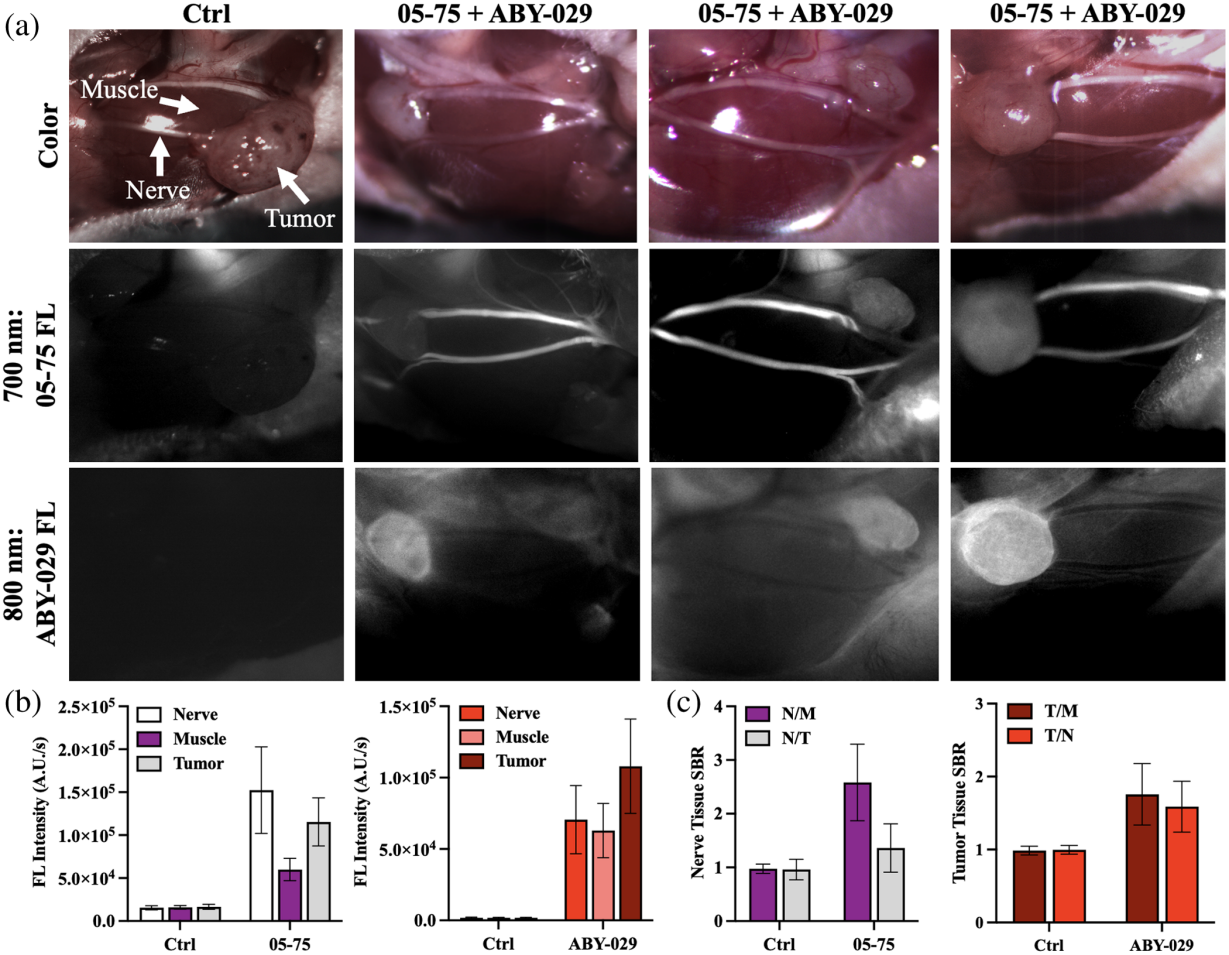
Co-administration of DSPE-PEG micelle–formulated LGW05-75 and ABY-029. (a) Representative images of Detroit 562 tumor–bearing athymic nude mice imaged in color and the optimal FL imaging channels to detect LGW05-75 (700 nm) and ABY-029 (800 nm) 4 h after systemic co-administration of vehicle (Ctrl, first column) or both NIR fluorescent probes (05-75 + ABY-029, second to fourth columns). (b) Quantified tissue fluorescence intensities of the nerve, muscle, and tumor tissues for both vehicle-injected control and fluorophore–administered mice in each fluorescence imaging channel (i.e., 700 nm = LGW05-75 and 800 nm = ABY-029) were used to calculate the (c) nerve and tumor SBR (N/M, N/T, T/M, and T/N) in their respective imaging channels (i.e., 700 nm = LGW05-75 and 800 nm = ABY-029).

The optimal nerve imaging time point following LGW05-75 administration was utilized to visualize murine nerve tissue in animals bearing head and neck tumors. To enable spectrally distinct visualization of the tumor tissue, mice were co-administered EGFR-targeted ABY-029 and nerve-specific LGW05-75. Four hours after systemic co-administration of DSPE-PEG micelle–formulated LGW05-75 and ABY-029, the tumor and facial nerves were exposed for fluorescence imaging. Images were collected with spectrally distinct excitation/emission filters centered at 620/700 nm for LGW05-75 (i.e., 700 nm) and 740/780 nm for ABY-029 (i.e., 800 nm) showing nerve and tumor specificity, respectively [Fig. 4(a)]. Nerve and tumor tissue fluorescence intensities were high in their respective imaging channels, whereas surrounding normal muscle tissue fluorescence intensity was low. Notably, nerve tissue fluorescence intensity was low in the ABY-029 imaging channel, and conversely, tumor tissue fluorescence intensity was low in the LGW05-75 imaging channel [Fig. 4(b)]. The tissue-specific accumulations of the LGW05-75 and ABY-029 probes in the nerve and tumor tissues, respectively, resulted in nerve-to-muscle and nerve-to-tumor tissue SBR>1.5 as well as tumor-to-muscle and tumor-to-nerve SBRs>1.5 [Fig. 4(c)].

**Fig. 5:**
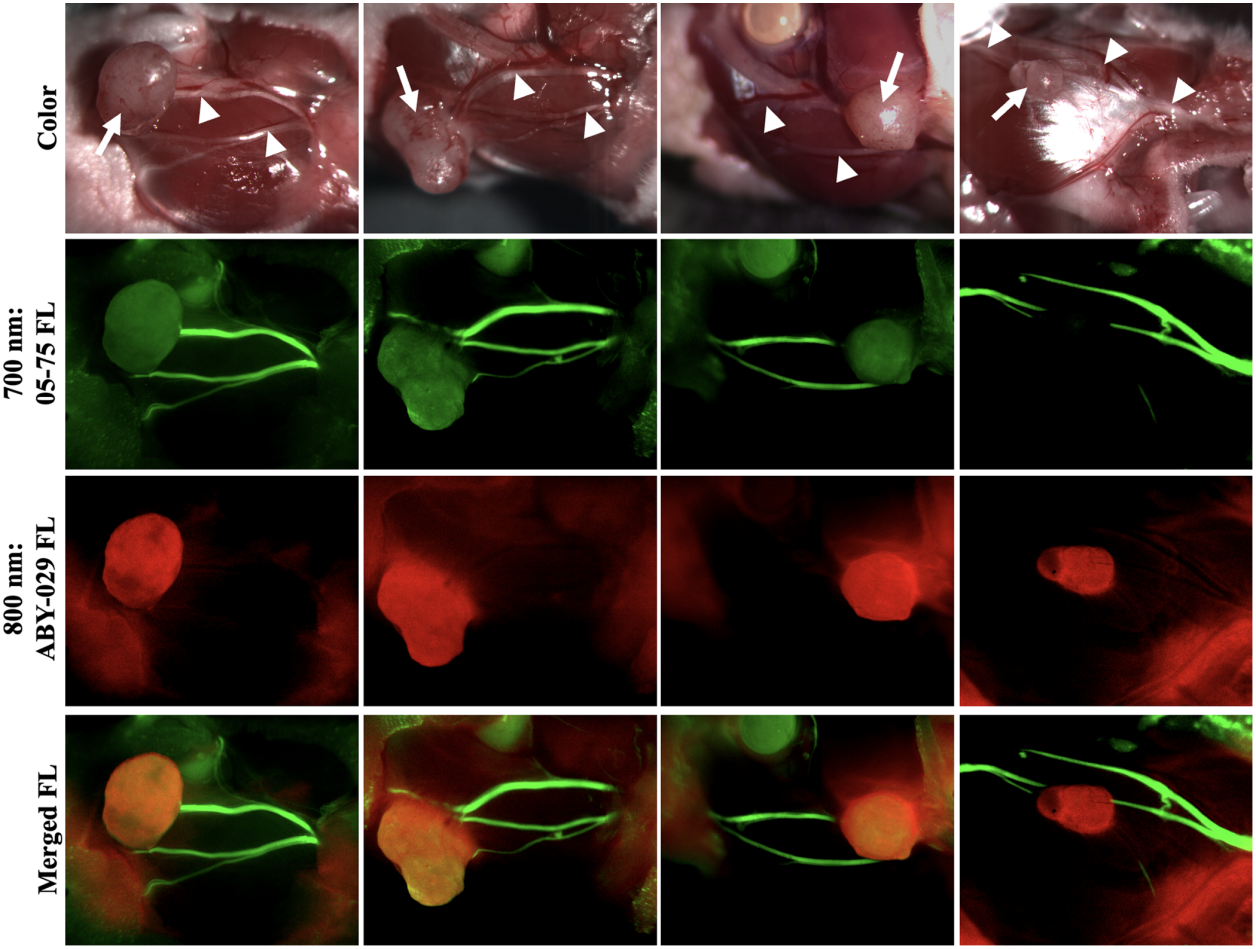
Two-color fluorescence nerve and tumor visualization. Representative images of athymic nude mice with Detroit 562 head and neck tumor xenografts 4 h after co-administration of DSPE-PEG-formulated LGW05-75 and ABY-029 co-administration. Images are shown in color (top), green false color for the 700-nm LGW05-75 imaging channel (second row), red false color for the 800-nm ABY-029 imaging channel (third row), and with the 700- and 800-nm false-colored fluorescence images merged (merged FL). The LGW05-75 highlighted the facial nerves, which are shown in the color image by the white arrowheads. The ABY-029 highlighted the Detroit 562 HNSCC tumor, which is shown in the color image by the white arrow.

The nerve-to-muscle contrast was not statistically different when LGW05-75 was administered alone or in combination with ABY-029 (P=0.14) to tumor-bearing mice. In addition, the nerve-to-tumor contrast was not statistically different when LGW05-75 was administered alone or in combination with ABY-029 (P=0.53) to tumor-bearing mice. Representative examples of multi-color fluorescence imaging false colored to visualize nerve and tumor tissue showed tissue-specific signal, where nerve tissue was highlighted by LGW05-75 and tumor tissue was highlighted by ABY-029, with a low accumulation of LGW05-75 in the tumor and some non-specific ABY-029 in surrounding normal tissues (Fig. 5).

## 4 Discussion and Conclusion

HNSCC remains a prevalent cancer diagnosis where surgical resection is the preferred treatment. However, successful completion of tumor resection and normal nerve tissue preservation remains challenging resulting in both positive surgical margins and lasting nerve damage. These complications lead to increased mortality due to cancer recurrence as well as increased morbidity due to lasting pain and loss of function. To improve surgical treatment for HNSCC, we have developed a two-color FGS strategy using spectrally distinct, NIR nerve- and tumor-specific fluorescent probes that permit co-administration for a complete intraoperative imaging solution. Herein, our focus was on the characterization of the PK profile of the NIR, nerve-specific fluorophore, LGW05-75, with the goal of enabling simultaneous co-administration with the tumor-targeted ABY-029 to enable seamless integration into the existing clinical workflow. The ability of the nerve- and tumor-targeted fluorescent probes to specifically highlight the targeted tissue with minimal accumulation in surrounding tissues as well as the ability to spectrally separate the fluorescence signals using conventional bandpass imaging systems was also evaluated to facilitate future clinical translation.

The PK profile of LGW05-75 for nerve-specific contrast was evaluated in both healthy CD-1 mice and athymic tumor-bearing nude mice, resulting in a similar conclusion that the 4-h time point after intravenous administration showed the highest nerve-specific contrast (Figs. 2 and 3). Fluorescent tissue intensities decreased at all measured time points after systemic administration; however, by the 4-h time point, non-specific muscle tissue fluorescence intensity had cleared from the tissue resulting in high nerve-to-muscle tissue contrast [Figs. 2(b), 2(c), 3(b), and 3(c)]. Notably, nerve tissue-specific fluorescence intensity at the 4-h time point was similar between the CD-1 non-tumor bearing mice and the athymic nude tumor-bearing mice, demonstrating minimal biodistribution effect of a tumor on nerve tissue accumulation from LGW05-75 [Figs. 2(b), 2(c), 3(b), and 3(c)]. Statistically significant nerve-to-background tissue contrast was present in the brachial plexus at all time points (i.e., 0.5, 1, 2, 4, and 6 h) after systemic administration in both normal and tumor-bearing animals, whereas statistically significant nerve-to-muscle contrast was present in the facial nerves 1 to 6 h after systemic administration in the normal mice and at all time points (i.e., 0.5, 1, 2, 4, and 6 h) after systemic administration in the tumor-bearing animals. This observed contrast difference was likely due to a combination of biological variance and the lower quantified nerve tissue fluorescence intensities in the small facial nerves (0.1 mm)^33^ compared with the larger diameter brachial plexus nerves (1 mm)^32^ in both the normal and tumor-bearing mice. Thus, the lower nerve tissue fluorescence intensities resulted in lower facial nerve SBR compared with the larger brachial plexus nerves as well as increased standard deviation among mouse imaging groups [Figs. 2(b) and 2(c)]. Importantly, nerve-specific contrast remained high in the facial nerves of both the normal and tumor-bearing animals at the optimal 4 h time point, where nerve SBR was >1.5, which is the published minimal SBR for clinically relevant contrast.^10^^–^^12^ In addition, the N/T SBR was found to be significant at the 2 and 4 h imaging time points [Fig. 3(c)]. These results demonstrate that LGW05-75 would provide strong nerve-to-background tissue contrast for the duration of an HNSCC surgical procedure and that the nerve-specific contrast agent had a matched PK profile to that of the published PK profile of tumor-targeted ABY-029 (Figs. 2 and 3).

To demonstrate the feasibility of two-color FGS for HNSCC, LGW05-75 and ABY-029 were co-administered to tumor-bearing mice. Similar nerve-specific fluorescence intensities were seen at the 4-h time point after systemic co-administration to that seen in the single LGW05-75 administration studies [Figs. 3(c) and 4(b)]. Variation in LGW05-75 uptake in tumors was seen across the tumor-bearing mouse cohort, where the nerve-to-tumor SBR was >1.5, demonstrating potential clinical utility [Fig. 4(c)]. Notably, there was also variation in ABY-029 uptake across tumors in the imaging cohort [Fig. 4(a)]. The variation in tumor tissue-specific fluorescence may be due to the differences in tumor vasculature content or could be model-dependent, which will be evaluated in future studies using additional xenograft and genetic mouse models. The feasibility of the two-color imaging approach to improve HNSCC surgery was further demonstrated in the representative false-colored images, where nerve tissue is depicted as green false color and tumor tissue is depicted as red false color, enabling a multicolor merged image for ready visualization of tissues to be resected (tumor) and tissues to be avoided (nerves, Fig. 5).

In summary, the studies herein show the feasibility of co-administration of nerve- and tumor-tissue targeted, NIR fluorophores permitting multi-color FGS to aid in HNSCC resections. Limitations of the current study include testing in a single tumor model, where additional HNSCC models will be utilized in the future to understand variability in ABY-029 uptake as well as any non-specific LGW05-75 accumulation. To enable future clinical translation, pharmacodynamic studies as well as co-administration pharmacology and toxicology profiles would need to be established. This work could also be readily extended to other cancer surgeries where tumor and nerve tissues reside in close proximity and optimal surgical decisions require consideration of both structures to minimize patient morbidity and mortality. The two-color FGS solution characterized herein will permit ready integration into the surgical workflow using nerve- and tumor-targeted agents with matching PK profiles that could be administered in the pre-operative arena or at the beginning of surgery permitting strong contrast in both tissues throughout a variety of surgical indications.

## Data Availability

All imaging data developed during the project will be made available upon reasonable request to the corresponding author, Summer Gibbs (gibbss@ohsu.edu).

## References

[1] Bray F. et al., “Global cancer statistics 2018: GLOBOCAN estimates of incidence and mortality worldwide for 36 cancers in 185 countries,” CA Cancer J. Clin. 68(6), 394–424 (2018). CAMCAM 0007-9235 10.3322/caac.21492 30207593

[2] Whitney M. A. et al., “Fluorescent peptides highlight peripheral nerves during surgery in mice,” Nat. Biotechnol. 30(4), 352–356 (2011). NABIF9 1087-0156 10.1038/nbt.1764 PMC3364105 21297616

[3] Chinn S. B. Myers J. N., “Oral cavity carcinoma: current management, controversies, and future directions,” J. Clin. Oncol. 33(29), 3269–3276 (2015). JCONDN 0732-183X 10.1200/JCO.2015.61.2929 26351335 PMC5320919

[4] Pfister D. G. et al., “Head and neck cancers, version 2.2020, NCCN clinical practice guidelines in oncology,” J. Natl. Comprehensive Cancer Netw. 18(7), 873–898 (2020). 10.6004/jnccn.2020.0031 32634781

[5] Aliperti L. A. et al., “Local and systemic recurrence is the Achilles heel of cancer surgery,” Ann. Surg. Oncol. 18(3), 603–607 (2011). 10.1245/s10434-010-1442-0 21161729 PMC11156256

[r6] Eldeeb H. et al., “The effect of the surgical margins on the outcome of patients with head and neck squamous cell carcinoma: single institution experience,” Cancer Biol. Med. 9(1), 29–33 (2012). 10.3969/j.issn.2095-3941.2012.01.005 23691451 PMC3643636

[7] Hinni M. L. et al., “Surgical margins in head and neck cancer: a contemporary review,” Head Neck 35(9), 1362–1370 (2013). 10.1002/hed.23110 22941934

[8] Woolgar J. A. Triantafyllou A., “A histopathological appraisal of surgical margins in oral and oropharyngeal cancer resection specimens,” Oral Oncol. 41(10), 1034–1043 (2005). EJCCER 1368-8375 10.1016/j.oraloncology.2005.06.008 16129652

[9] McMahon J. et al., “Influence of condition of surgical margins on local recurrence and disease-specific survival in oral and oropharyngeal cancer,” Br. J. Oral Maxillofac. Surg. 41(4), 224–231 (2003). 10.1016/S0266-4356(03)00119-0 12946663

[10] Baxi S. S. et al., “Causes of death in long-term survivors of head and neck cancer,” Cancer 120(10), 1507–1513 (2014). CANCAR 0008-543X 10.1002/cncr.28588 24863390 PMC4101810

[r11] London J. London N. J. Kay S. P., “Iatrogenic accessory nerve injury,” Ann. R. Coll. Surg. Engl. 78(2), 146–150 (1996). ARCSAF 0035-8843 8678450 PMC2502542

[r12] Boström D. Dahlin L. B., “Iatrogenic injury to the accessory nerve,” Scand. J. Plastic Reconstr. Surg. Hand Surg. 41(2), 82–87 (2009). 10.1080/02844310600836810 17605441

[r13] Pai P. S., “Complications in head and neck surgery,” Int. J. Otorhinolaryngol. Clin. 2(1), 61–67 (2010). 10.5005/jp-journals-10003-1018

[14] Poh C. F. et al., “Canadian Optically-guided approach for Oral Lesions Surgical (COOLS) trial: study protocol for a randomized controlled trial,” BMC Cancer 11, 462 (2011). BCMACL 1471-2407 10.1186/1471-2407-11-462 22026481 PMC3226575

[15] Chua K. S. G. et al., “Pain and loss of function in head and neck cancer survivors,” J. Pain Symptom Manage. 18(3), 193–202 (1999). 10.1016/S0885-3924(99)00070-6 10517041

[16] de Bree R. Leemans C. R., “Recent advances in surgery for head and neck cancer,” Curr. Opin. Oncol. 22(3), 186–193 (2010). CUOOE8 1040-8746 10.1097/CCO.0b013e3283380009 20173640

[17] Barth C. W. Gibbs S. L., “Fluorescence image-guided surgery—a perspective on contrast agent development,” Proc. SPIE 11222, 112220J (2020). PSISDG 0277-786X 10.1117/12.2545292 PMC7115043 32255887

[18] Azari F. et al., “Prospective validation of tumor folate receptor expression density with the association of pafolacianine fluorescence during intraoperative molecular imaging-guided lung cancer resections,” Eur. J. Nucl. Med. Mol. Imaging 50(8), 2453–2465 (2023). 10.1007/s00259-023-06141-3 36905412 PMC10314365

[r19] Hwang E. S. et al., “Clinical impact of intraoperative margin assessment in breast-conserving surgery with a novel pegulicianine fluorescence-guided system: a nonrandomized controlled trial,” JAMA Surg. 157(7), 573–580 (2022). 10.1001/jamasurg.2022.1075 35544130 PMC9096689

[r20] Sarkaria I. S. et al., “Pafolacianine for intraoperative molecular imaging of cancer in the lung: the ELUCIDATE trial,” J. Thorac. Cardiovasc. Surg. 166(6), e468–e478 (2023). JTCSAQ 0022-5223 10.1016/j.jtcvs.2023.02.025 37019717 PMC12507096

[r21] Smith B. L. et al., “Intraoperative fluorescence guidance for breast cancer lumpectomy surgery,” NEJM Evid. 2(7), EVIDoa2200333 (2023). 10.1056/EVIDoa2200333 38320161

[22] Tanyi J. L. et al., “A phase III study of pafolacianine injection (OTL38) for intraoperative imaging of folate receptor-positive ovarian cancer (study 006),” J. Clin. Oncol. 41(2), 276–284 (2023). JCONDN 0732-183X 10.1200/JCO.22.00291 36070540 PMC12684809

[23] Wang L. G. et al., “Near-infrared nerve-binding fluorophores for buried nerve tissue imaging,” Sci. Transl. Med. 12(542), eaay0712 (2020). STMCBQ 1946-6234 10.1126/scitranslmed.aay0712 32376766

[24] Gibbs-Strauss S. L. et al., “Nerve-highlighting fluorescent contrast agents for image-guided surgery,” Mol. Imaging 10(2), 91–101 (2011). 10.2310/7290.2010.00026 21439254 PMC4386639

[25] Barth C. W. et al., “Nerve-sparing gynecologic surgery enabled by a near-infrared nerve-specific fluorophore using existing clinical fluorescence imaging systems,” Small, e2300011 (2023). SMALBC 1613-6810 10.1002/smll.202300011 37452434 PMC11042870

[26] Barth C. W. et al., “Clinically translatable formulation strategies for systemic administration of nerve-specific probes,” Adv. Ther. 4(7), 2100002 (2021). 10.1002/adtp.202100002 PMC8372234 34423111

[27] Samkoe K. S. et al., “Toxicity and pharmacokinetic profile for single-dose injection of ABY-029: a fluorescent anti-EGFR synthetic Affibody molecule for human use,” Mol. Imaging Biol. 19(4), 512–521 (2017). 10.1007/s11307-016-1033-y 27909986 PMC5648591

[r28] Samkoe K. S. et al., “Preclinical imaging of epidermal growth factor receptor with ABY-029 in soft-tissue sarcoma for fluorescence-guided surgery and tumor detection,” J. Surg. Oncol. 119(8), 1077–1086 (2019). JSONAU 0022-4790 10.1002/jso.25468 30950072 PMC6529257

[29] Wang C. et al., “Improved discrimination of tumors with low and heterogeneous EGFR expression in fluorescence-guided surgery through paired-agent protocols,” Mol. Imaging Biol. 25(1), 110–121 (2023). 10.1007/s11307-021-01656-3 34651290 PMC9527767

[30] Barth C. W. et al., “A clinically relevant formulation for direct administration of nerve specific fluorophores to mitigate iatrogenic nerve injury,” Biomaterials 284, 121490 (2022). BIMADU 0142-9612 10.1016/j.biomaterials.2022.121490 35395454 PMC9064958

[r31] Barth C. W. et al., “Lead optimization of nerve-specific fluorophores for image-guided nerve sparing surgical procedures,” in Opt. Mol. Probes Imaging Drug Deliv., p. OW3E.3 (2021). 10.1364/OMP.2021.OW3E.3 PMC9431774 36053248

[32] Wang L. G. et al., “Nerve visualization using phenoxazine-based near-infrared fluorophores to guide prostatectomy,” Adv. Mater. 36(16), e2304724 (2023). ADVMEW 0935-9648 10.1002/adma.202304724 37653576

[33] Wanner R. et al., “Three-dimensional in vivo magnetic resonance imaging (MRI) of mouse facial nerve regeneration,” Front. Neurol. 10, 310 (2019). 10.3389/fneur.2019.00310 31001195 PMC6454117

